# N-domain of angiotensin-converting enzyme hydrolyzes human and rat amyloid-β(1-16) peptides as arginine specific endopeptidase potentially enhancing risk of Alzheimer’s disease

**DOI:** 10.1038/s41598-017-18567-5

**Published:** 2018-01-10

**Authors:** Elena V. Kugaevskaya, Alexander V. Veselovsky, Maria I. Indeykina, Nina I. Solovyeva, Maria S. Zharkova, Igor A. Popov, Eugene N. Nikolaev, Alexey B. Mantsyzov, Alexander A. Makarov, Sergey A. Kozin

**Affiliations:** 1 Institute of Biomedical Chemistry, Moscow, Russia; 2 Engelhardt Institute of Molecular Biology of the Russian Academy of Sciences, Moscow, Russia; 3 Emanuel Institute of Biochemical Physics of the Russian Academy of Sciences, Moscow, Russia; 4 Moscow Institute of Physics and Technology, Dolgoprudnyi, Moscow Region Russia; 5 Skolkovo Institute of Science and technology, Moscow, Russia; 6 Faculty of Fundamental Medicine, Lomonosov Moscow State University, Moscow, Russia

## Abstract

Alzheimer’s disease (AD) is a multifactorial neurodegenerative disorder. Amyloid-β (Aβ) aggregation is likely to be the major cause of AD. In contrast to humans and other mammals, that share the same Aβ sequence, rats and mice are invulnerable to AD-like neurodegenerative pathologies, and Aβ of these rodents (ratAβ) has three amino acid substitutions in the metal-binding domain 1-16 (MBD). Angiotensin-converting enzyme (ACE) cleaves Aβ-derived peptide substrates, however, there are contradictions concerning the localization of the cleavage sites within Aβ and the roles of each of the two ACE catalytically active domains in the hydrolysis. In the current study by using mass spectrometry and molecular modelling we have tested a set of peptides corresponding to MBDs of Aβ and ratAβ to get insights on the interactions between ACE and these Aβ species. It has been shown that the N-domain of ACE (N-ACE) acts as an arginine specific endopeptidase on the Aβ and ratAβ MBDs with C-amidated termini, thus assuming that full-length Aβ and ratAβ can be hydrolyzed by N-ACE in the same endopeptidase mode. Taken together with the recent data on the molecular mechanism of zinc-dependent oligomerization of Aβ, our results suggest a modulating role of N-ACE in AD pathogenesis.

## Introduction

Amyloid-β (Aβ) is a 39–43 amino acid long peptide heterogenic at the C-terminus (Aβ(1–39 … 43)) and a normal component of biological fluids of humans and other mammals at picomolar concentration levels^1^. In Alzheimer’s disease (AD) endogenous Aβ converts to soluble neurotoxic oligomers^2^ and accumulates as insoluble extracellular aggregates (amyloid plaques) in the brain tissue^3^. According to the amyloid cascade hypothesis, which has been the predominant framework for A D studies, Aβ aggregation plays a unique and critical role as the initiator of the pathology^4,5^. What triggers Aβ aggregation still remains unclear, however, some genetically and/or post-translationally modified Aβ species accumulated in the amyloid plaques appear to act as pathogenic aggregation seeds^6^. For example, such role in AD amyloidogenesis has been proposed for N-truncated Aβ species generated from hydrolysis by arginine endopeptidases^7^.

Many factors appear to accelerate AD, cerebrovascular disease being the foremost among them^5,8^. Hypertension is one of the major modifiable risk factors for cognitive decline in the elderly that can lead to AD^9–13^. Meta-analysis of studies investigating the ability of antihypertensive drugs to prevent age-related dementia show results, suggesting a beneficial effect^14^. In clinical practice one of the main hypertension treatment methods is based on the use of angiotensin converting enzyme (ACE) inhibitors^15^. ACE (peptidyl-dipeptidase A, EC 3.4.15.1) is the key enzyme of the renin–angiotensin and kallikrein–kinin systems responsible for the regulation of blood pressure and electrolyte homeostasis^16^. Usually ACE acts as a dipeptidyl carboxypeptidase that catalyzes the hydrolytic cleavage of dipeptides from the carboxyl terminus of a wide variety of oligopeptides^17^. Somatic ACE is a membrane-bound zinc metalloprotease composed of two homologous catalytic N- and C-domains whose sequences share 60% of identity, but in the regions involved in catalysis homology reaches 89%^18^. Crystallographic data suggests that although the overall spatial structures of the N- and C-domains are very similar, their active sites are quite different, and this seems to determine the substrate specificity of the domains^19,20^.

Early indications that the ACE gene may have some relevance to AD came from studies showing that ACE activity is increased in the AD brain, especially in the hippocampus and frontal cortex where amyloid plaques are most abundant^21^. Additional supportive evidence of the role of ACE in AD comes from findings of increased ACE activity in postmortem AD brain tissues, in direct relation to parenchymal Aβ load^22^ and Braak-staged AD severity^23^. Two independent groups reported that a relatively common insertion/deletion polymorphism in the ACE gene was associated with late-onset AD in a number of population studies^24,25^. These observations were later supported by a subsequent deep meta-analysis study^26^. The significance of ACE for AD pathogenesis may be due to specific hydrolysis of Aβ by ACE^27–32^. There is some epidemiological evidence indicating that brain-penetrating ACE inhibitors (ACE-Is) may slow the risk of cognitive decline^33–37^. ACE-Is have shown positive effects on cognition in various AD models^38,39^. Treatment with a centrally active ACE inhibitor, captopril, slows Aβ plaque accumulation in the hippocampus of AD mice^40^, thus suggesting that cognitive amelioration caused by ACE-Is is linked to the suppression of Aβ aggregation. But the molecular mechanisms responsible for these protective effects of antihypertensive drugs have not yet been identified^41^.

Taking into account that ACE does not participate in the regulation of steady-state Aβ levels in the brain^42^ we have hypothesized a role of Aβ species processed by the N-domain of ACE at the Arg5-His6 bond as aggregation seeds for endogenous Aβ^43^. Unfortunately, there is an inconsistency concerning the exact localization of the cleavage sites within Aβ upon ACE hydrolysis^27–32^. Current data also provides conflicting information on whether the active site of the N- or the C-domain participates in Aβ proteolysis, and whether ACE acts as an endopeptidase or a carboxypeptidase. All these uncertainties probably come from non-optimal peptide substrates used in the studies. Specifically, Aβ peptide substrates intended for testing endoproteolytical activity of ACE should be C-amidated in order to better represent the situation when the peptide forms N-terminal part of a longer polypeptide chain (as in the case of Aβ(1-40) or Aβ(1-42)). Since the majority of the reported ACE cleavage sites^27–32^ are located in the Aβ N-terminal metal-binding domain 1DAEFRHDSGYEVHHQK16 (MBD)^44–48^, the synthetic MBD analogs with intact or modified N- and C-terimini would serve as adequate experimental ACE substrates. Notably, the three amino acid substitutions (Arg5Gly, Tyr10Phe, and His13Arg) distinguishing human amyloid-β (Aβ) from that of rats and mice (ratAβ), who are invulnerable to AD-like neurodegenerative pathologies in contrast to other mammals^49,50^ are located in the MBD. In the current work using mass-spectrometry and molecular modelling we have tested a set of synthetic peptides (with free, as well as partially or fully protected termini) corresponding to Aβ MBD and ratAβ MBD (Table 1, Supplementary Fig. S1) as substrates for N- and C- domains of ACE to get more insights into the role of the interactions between ACE and Aβ in AD pathogenesis.

**Table 1:** Calculated and observed [M + H^+^] ions of synthetic analogs of Aβ and ratAβ metal-binding domains and their cleavage products generated by the action of N-ACE and C-ACE.

Amyloid peptide	Peptide sequence	Calculated m/z	Mean Observed m/z
**Substrates**			
Aβ(1-16)	DAEFRHDSGYEVHHQK	1954.87906	1954.8
Aβ(1-16)-[Amide]	DAEFRHDSGYEVHHQK-[Amide]	1953.89505	1953.9
[Acetyl]-Aβ(1-16)-[Amide]	[Acetyl]- DAEFRHDSGYEVHHQK Amide]	1995.90561	1995.8
[Acetyl]-ratAβ(1-16)-[Amide]	[Acetyl]-DAEFGHDSGFEVRHQK-[Amide]	1899.87325	1899.9
**Products**			
Aβ(1-14)	DAEFRHDSGYEVHH	1698.72552	1698.6
Aβ(1-13)	DAEFRHDSGYEVH	1561.66661	1561.5
Aβ(6-16)-[Amide]	HDSGYEVHHQK-[Amide]	1335.61887	1335.6
[Acetyl]-Aβ(1-5)	[Acetyl]- DAEFR	679.30458	679.3
[Acetyl]-ratAβ(1-15)	[Acetyl]-DAEFGHDSGFEVRHQ	1772.76230	1772.9
[Acetyl]-ratAβ(1-14)	[Acetyl]-DAEFGHDSGFEVRH	1644.70372	1644.9
[Acetyl]-ratAβ(1-13)	[Acetyl]-DAEFGHDSGFEVR	1507.64481	1507.5

## Results and Discussion

### C-terminal amidation switches the cleavage mechanism of N-ACE towards Aβ(1-16) species from unspecific carboxypeptidase action to arginine specific endopeptidase

Earlier we have shown that a model synthetic [Acetyl]-Aβ(1-16)-[Amide] peptide, both ends of which are protected, is hydrolyzed only by the N-domain of ACE, which cleaves the Arg5-His6 bond, while the C-domain does not affect any of the bonds in this peptide^32^. Other researchers using peptides with unprotected ends have shown that the hydrolysis of Aβ(1–16) by both domains of ACE is not limited or specific, and that under certain conditions the C-domain also hydrolyzes Aβ peptides^29^.

In the present study, in order to determine the effect of termini protection on the hydrolysis of Aβ metal-binding domain (MBD) by the N-domain of АСЕ (N-АСЕ), we studied the interaction of N-АСЕ with three peptides: Аβ(1-16), Aβ(1-16)-[Amide] and [Acetyl]-Aβ(1-16)-[Amide] (Table 1). Each peptide (40 μM) was incubated in two different buffer systems (see the section 2.4.) at 37 °C with N-ACE for 10–40 min. Additionally, these reactions were performed in the presence of lisinopril (10 μM) known as a specific inhibitor of ACE enzymatic activity. Samples from all of the reaction mixtures were subjected to direct MALDI-TOF MS analysis in order to identify the reaction products.

Mass spectrum of Аβ(1-16), incubated for 40 min with N-ACE in the bicarbonate buffer system, is shown in Supplementary Fig. S2. Besides the peak corresponding to the parent peptide molecular ion (m/z 1954.8), there is another significant peak with m/z value 1698.6 which is characteristic for the Аβ(1-14) peptide (Table 1). The dipeptide Gln15-Lys16 due to its low mass falls into the matrix suppression region and is, thus, not observed in the mass-spectrum. So, in case of Аβ(1-16) with free N- and C-termini, N-АСЕ acts as a carboxydipeptidase, by cleaving the Нis14-Gln15 bond (Table 2), what is in good agreement with the results presented by Larmuth *et al*. on the hydrolysis of Аβ(1-16) by various forms of recombinant АСЕ^29^.

**Table 2:** Hydrolysis of Aβ peptides under study by the ACE N- and C- domains.

Enzyme	Products observed (backbone positions)		Bond cleaved	
Substrate	N-ACE	C-ACE	N-ACE	C-ACE
Aβ(1–16)	1–14	1–13, 1–14	13–14	13–14, 15–15
Aβ(1–16)-[Amide]	6–16	ND*	5–6	ND
[Acetyl]-Aβ(1–16)-[Amide]	1–5, 6–16	ND	5–6	ND
[Acetyl]-ratAβ(1–16)-[Amide]	1–13	1–13, 1–14, 1–15	13–14	13–14, 14–15, 15–16

*Not detected (ND).

The mass spectra obtained from the reaction mixture, wherein Aβ(1-16)-[Amide] and [Acetyl] -Aβ(1-16)-[Amide] had been incubated for 130 min with N-ACE in the bicarbonate buffer, are shown in Supplementary Fig. S2. For both reaction mixtures, signals of respective parent peptide molecular ions (m/z 1953.9 and 1995.8) are accompanied by a peak (m/z 1335.6) corresponding to the Aβ(6-16)-[Amide] peptide (Table 1). In the [Acetyl]-Aβ(1-16)-[Amide]/N-ACE reaction mixture the complementary peak (m/z 679.3) attributed to [Acetyl]-Aβ(1-5) is also detected (data no shown). The specificity of N-ACE activity has been confirmed by complete inhibition of hydrolysis by the ACE inhibitor lisinopril (Supplementary Fig. S2). Thus in contrast to Aβ(1-16), N-ACE cleaves Aβ(1-16)-[Amide] and [Acetyl]-Aβ(1-16)-[Amide] at the Arg5-His6 site, therefore, acting for these substrates as an endopeptidase (Table 2).

**Figure 1:**
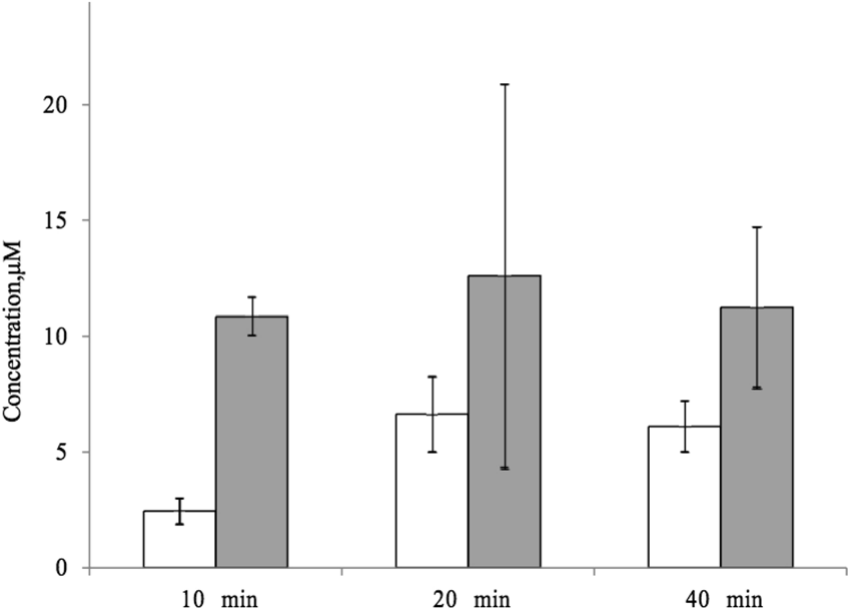
Concentrations of Aβ(6-16)-[Amide] in the reaction mixtures wherein 20 μM of [Acetyl]-Aβ(1-16)-[Amide] (white boxes) or 20 μM of Aβ(1-16)-[Amide] (grey boxes) were incubated for 10-40 min with N-ACE.

In order to determine whether acetylation of the N-terminus affects the efficiency of the Arg5-His6 bond cleavage in the Aβ MBD, a second series of experiments was carried out, in which the Arg5-His6 cleavage efficiency was compared for peptides Aβ(1-16)-[Amide] and [Acetyl]-Aβ(1-16)-[Amide]. For this in each of the two reaction mixtures, containing one of the peptides and N-ACE, the amount of one of the two products of hydrolysis – Aβ(6-16)-[Amide] – was monitored using ^18^O-labeled internal standards as described earlier^32,51^. Briefly, the absolute peptide concentrations of the reaction products in respective mixtures were calculated by employing a linear correlation between the peak height ratio and sample load. Thus it was shown that the amount of Aβ(6-16)-[Amide], formed from enzymatic cleavage of Aβ(1-16)-[Amide] was 1.7–4 times higher than from the cleavage of [Acetyl]-Aβ(1-16)-[Amide] (Fig. 1). Despite the fact that Aβ(1-16)-[Amide] (with the free N-terminal aminogroup) is more efficiently cleaved by N-ACE than [Acetyl]-Aβ(1-16)-[Amide], both peptides are cleaved at the same site (Arg5-His6). Thus addition of an acetyl protective group to the N-terminus of the Aβ(1-16)-[Amide] peptide decreases the efficiency of hydrolysis by N-ACE, but does not affect the specificity of N-ACE, which acts as an endopeptidase on both peptides. Altogether, our data shows that N-АСЕ acts as a specific endopeptidase only towards Аβ MBD species with a C-terminal blocking amide group, while protection of the N-terminus of these peptides does not change the specificity of hydrolysis by N-АСЕ (Table 2).

### C-terminal amidation blocks C-ACE action on Aβ(1-16) species

In contrast to N-ACE, the C-domain of ACE under the same experimental conditions does not cleave neither Aβ(1-16)-[Amide] nor [Acetyl]-Aβ(1-16)-[Amide] at any peptide bond as evidenced by MALDI-TOF mass spectra of respective reaction mixtures. In these spectra only the parent molecular ions (m/z 1953.9 and 1995.8) are observed (Supplementary Fig. S3). When incubated with Аβ(1-16), besides the parent molecular ion (m/z 1954.8) peaks with m/z 1561.5 and m/z 1698.6, corresponding to fragments Аβ(1-13) and Аβ(1-14) respectively are present in the spectra (Supplementary Fig. S3). This indicates that С-АСЕ hydrolyses the Нis14-Gln15 and Нis13-Нis14 bonds within Аβ(1-16), forming С-terminal di- and tripeptides, and so acts as a carboxypeptidase (Tables 1 and 2). The specificity of this reaction was validated by a parallel experiment in the presence of lisinopril, whose presence fully prevented the formation of these products of hydrolysis (Supplementary Fig. S3).

That C-ACE acts as a carboxypeptidase towards Аβ(1-16), was also shown earlier^29^ and is in good agreement with the well-known properties of ACE, which mostly cleaves C-terminal dipeptides from oligopeptides with a free carboxylic group^17^. Here for the first time we have shown that addition of a blocking amide group to the C-end of Аβ(1-16) completely prevents the resulting peptide Aβ(1-16)-[Amide] from being hydrolyzed by С-АСЕ (Supplementary Fig. S3). The peptide [Acetyl]-Aβ(1-16)-[Amide], which besides the amide protective group at the N-terminus carries an acetyl protective group at the C-end, also is not cleaved by С-АСЕ (Supplementary Fig. S3), what is in good agreement with our previous observations^32^.

### [Acetyl]-ratAβ(1-16)-[Amide] is cleaved specifically at the Arg-His bond by N-ACE and unspecifically at the C-terminus by C-ACE

As shown above N-ACE specifically cleaves the Arg5-His6 bond in C-amidated analogs of the metal-binding domain of human amyloid-β (Aβ). Metal-binding domains (MBDs) of Aβ and ratAβ differ by three amino acid substitutions (Arg5Gly, Tyr10Phe, His13Arg). Due to these substitutions the ratAβ MBD lacks the Arg5-His6 site, and an alternative site Arg13-His14 is formed. Considering, that C-terminal amidation of Aβ MBD is necessary for endopeptidase activity of N-ACE towards this peptide, аnd N-terminal acetylation does not affect the products of hydrolysis of Aβ MBD by neither N-ACE nor C-ACE (see sections 2.1. and 2.2.), we used a synthetic peptide [Acetyl]-ratAβ(1-16)-[Amide] as a model substrate to study the proteolysis of ratAβ MBD by N- and C-ACE domains.

Mass spectrum of the peptide [Acetyl]-ratAβ(1-16)-[Amide], incubated for 60 min with N-ACE in the bicarbonate buffer, is shown in Supplementary Fig. S4. Besides the peak corresponding to the parent peptide molecular ion (m/z 1899.9), another significant peak with m/z 1507.5 which is characteristic for the [Acetyl]-Аβ(1-13) peptide is observed (Table 1). After incubation of [Acetyl]-ratAβ(1-16)-[Amide] with C-ACE, peaks corresponding to ratAβ MBD (m/z 1899.9), [Acetyl]-ratАβ(1-13) (m/z 1507.5), [Acetyl]-ratАβ(1-14) (m/z 1644.9), and [Acetyl]-ratАβ(1-15) (m/z 1772.9) have been registered (Tables 1 and 2, Supplementary Fig. S4). The specificity of N-ACE and C-ACE activities have been confirmed by complete inhibition of hydrolysis by the ACE inhibitor, lisinopril (Supplementary Fig. S4). This indicates that N-АСЕ hydrolyses only one single bond Arg13-His14 in [Acetyl]-ratAβ(1-16)-[Amide], while С-АСЕ cleaves this peptide in three locations: Arg13-His14, His14-Gln15, and Gln15- Lys16 (Table 2).

**Figure 2:**
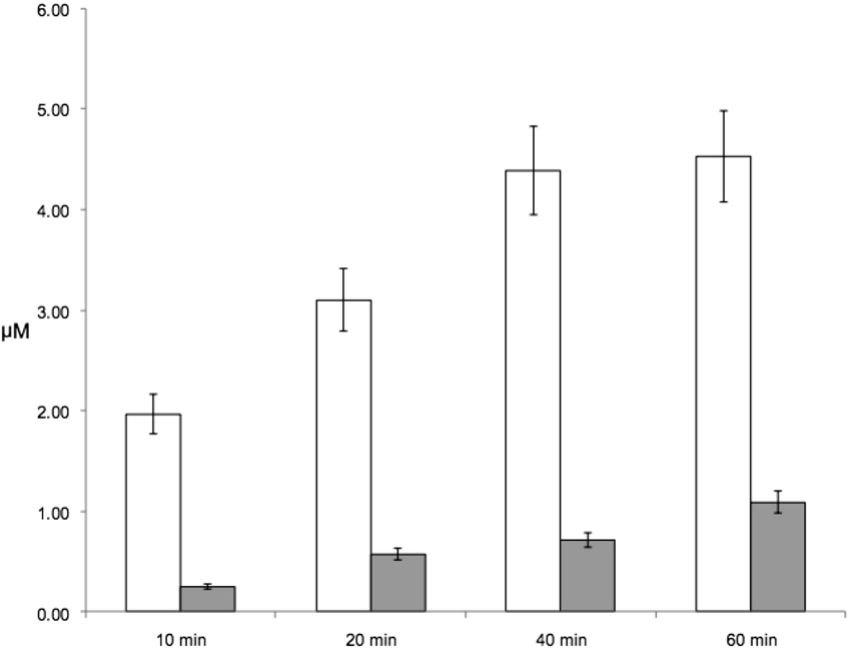
Concentrations of [Acetyl]-ratAβ(1-13) in the reaction mixtures wherein 20 μM of [Acetyl]-ratAβ(1-16)-[Amide] were incubated for 60 min with N-ACE (white boxes) or with C-ACE (grey boxes).

To evaluate the efficiency of the Arg13-His14 peptide bond cleavage of [Acetyl]-ratAβ(1-16)-[Amide] by N-ACE in comparison with C-ACE, quantitation of digestion products was performed by direct MALDI-TOFMS using ^18^O-labeled internal standards as described earlier^32,51^. The isotopic patterns corresponding to the unlabeled [Acetyl]-ratAβ(1-13), ^18^O labeled standard of [Acetyl]-ratAβ(1-13), and the analyte/standard mixtures of interest are shown in Supplementary Fig. S5. The absolute peptide concentrations of the resulting reaction product were calculated from the intensity ratios of the non-labeled peptide peaks and those of the labeled standard. It was shown that the amount of [Acetyl]-ratAβ(1-13), formed from enzymatic cleavage of [Acetyl]-ratAβ(1-16)-[Amide] by N-ACE was 4–4.5 times higher than from the reaction with C-ACE (Fig. 2). So, N-ACE hydrolyses the Arg13-His14 bond of [Acetyl]-ratAβ(1-16)-[Amide] much more efficiently than C-ACE does. Thus basing on this data it can be concluded that similarly to human Aβ, N-ACE cleaves [Acetyl]-ratAβ(1-16)-[Amide] specifically at the Arg-His bond and C-ACE does so unspecifically at the C-terminus acting as a carboxypeptidase.

### Molecular modeling of complexes of Аβ-derived substrates with the active center of N-ACE supports the role of N-ACE as an arginine endopeptidase towards Аβ species

We have shown that N-АСЕ demonstrates endoproteolytic activity by cleaving the Arg-His bond in C-amidated Аβ and ratАβ MBDs irrelevant of the bond position whether 5–6 (in human) or 13–14 (in rat). To get more insight into the molecular mechanism of N-ACE endoproteolytical activity, the complexes of N-ACE with tetrapeptides corresponding to several fragments of Аβ and ratАβ MBDs have been modelled.

The active site of N-ACE (the structure of the C-domain of ACE is very similar) is a large channel with a constriction in the middle, which divides the channel into two chambers like in a sand-glass with a catalytic Zn^2+^ in the center^52^. The active site is quite large and can accommodate several amino acids in both parts. Since it is difficult to correctly model the complexes of N-ACE with long peptides (Аβ(1-16) or ratАβ(1-16)), in this study tetrapeptides 4FRHD7 (h4_7) and 12VHHQ15 (h12_15) of Аβ and 4FGHD7 (r4_7) and 12VRHQ15 (r12_15) of ratАβ have been used to model the behaviour of (Аβ(1-16) and ratАβ(1-16) as substrates for N-ACE. We have implemented molecular dynamic simulation to probe the stability of Michaelis complexes for N-ACE with h4_7, h12_15, r4_7, and r12_15 substrates in the N-ACE active site and figure out the possible reasons for the abolishment of catalytic activity, associated with R5G, Y10F, and H13R substitutions by which Аβ differs from ratАβ^50^.

**Figure 3:**
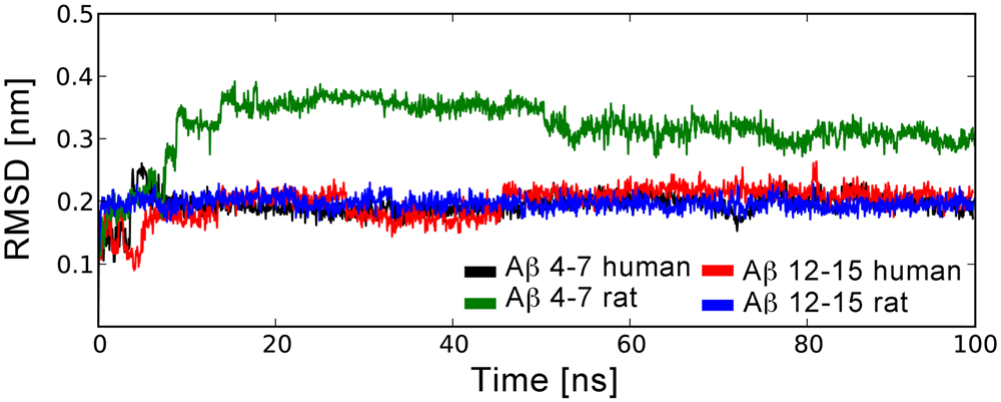
Fluctuations of the RMSD values for the Aβ tetrapeptidic fragments bound at N-ACE active site along the molecular dynamic trajectories. RMSD values were calculated over all peptide atoms relative to the initial structures.

**Figure 4:**
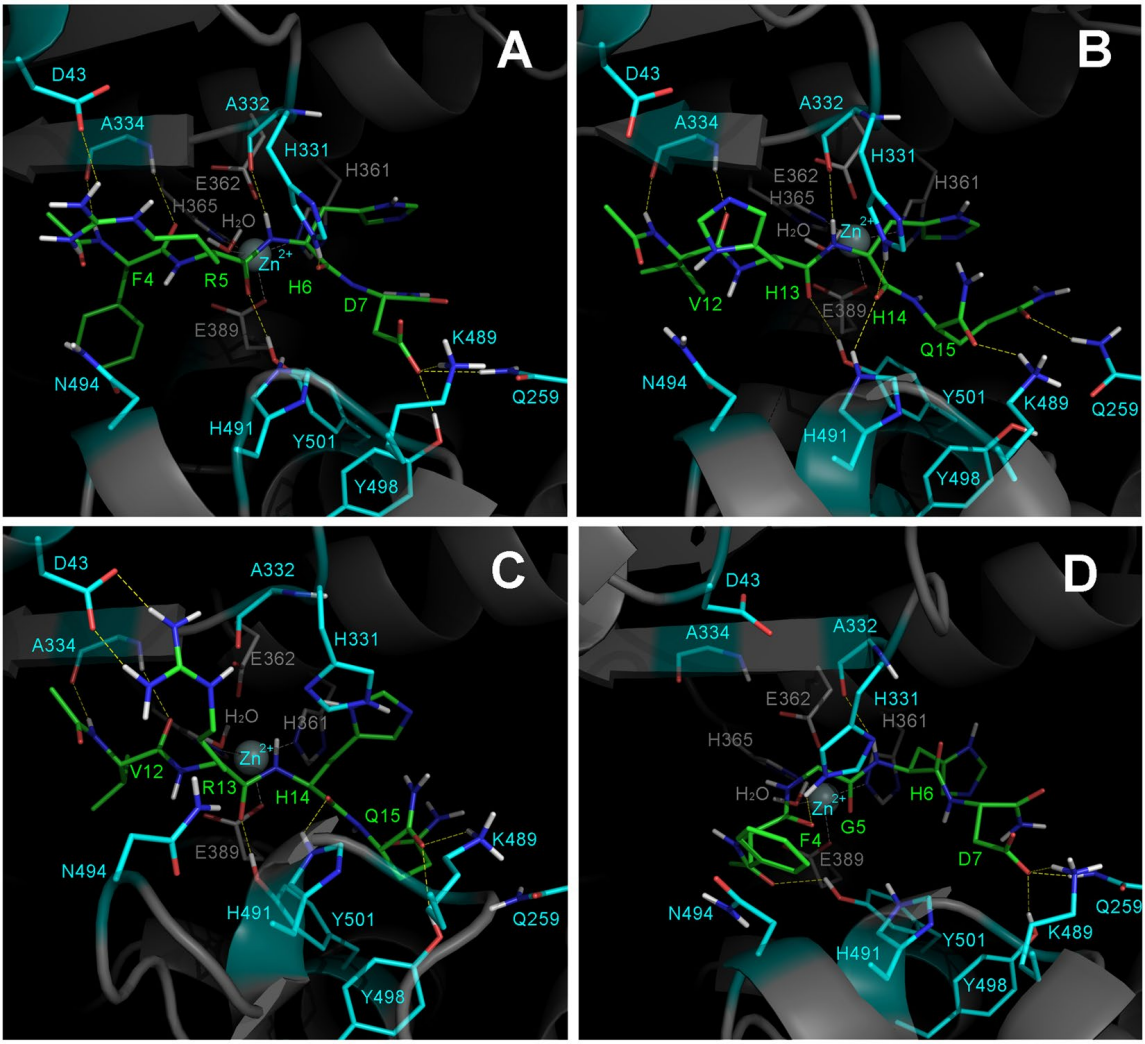
Snapshots from the molecular dynamic trajectories representing the statistically significant hydrogen bonds for the h4_7 (**A**), h12_15 (**B**), and r12_15 (**C**) Aβ tetrapeptides bound at N-ACE substrate tunnel. Panel D shows distorted conformation of r4_7 at the final time step of the molecular dynamic trajectory, where both characteristic hydrogen bonds F4 O - H331 Nε2 Hε2 and N-terminal acetyl O - Y501 OχHχ are observed. Peptide carbon atoms are in green; carbon atoms of receptor residues, important for peptide stabilization, are in cyan; grey sticks depict receptor residues, involved in zinc chelation and catalysis.

All four systems were stable along the course of the 100 ns molecular dynamic simulation and the tetrahedral zinc coordination has been retained (Supplementary Table S3). Peptides h4_7, h12_15 and r12_15 have demonstrated similar conformational behavior and interactions with the N-ACE active site (Figs 3 and 4A–C, Supplementary Table S5). These three tetrapeptides adopted an extended backbone conformation, which has been stabilized by hydrogen bonds with main-chain atoms of β-sheet N-ACE residues A332 and A334 and side chains of H331, H491 and Y501. This behaviour is in line with the experimental and theoretical studies of ACE complexes with known peptide substrates^53–55^. Constructs h4_7 and r12_15 demonstrate more than 89% populations of the key contacts, stabilizing the scissile bond (R5 O – Y501 OχHχ, H6 NH – A332 O for h4_7 and R13 O – Y501 OχHχ, H14 NH – A332 O for r12_15, Supplementary Table S5). The h12_15 construct reveals ca. 30% decrease of the H13 O – Y501 OχHχ contact population as compared to h4_7 and r12_15. The side chain of an arginine residue, preceding the scissile bond, interacted with the carboxyl group of D43 and amide group of N494 of N-ACE. Polar groups of C-terminal amino acids of h4_7, h12_15 and r12_15 formed hydrogen bonds with N-ACE residues Q259, K489 and Y498. The hydrogen bond, linking the side chain of the N-ACE catalytic residue E362 and zinc-coordinating water molecule, was stable along the whole simulation. It is interesting to note, that higher flexibility of the arginine side chain, preceding the scissile bond, as compared to the bulky histidine imidazole ring, results in the weaker stabilization of the N-terminus and decreased populations of the H6 O– H331 Nε2 Hε2 and H14 O – H331 Nε2 Hε2 contacts for h4_7 and r12_15 peptides respectively as compared to analogous contacts of h12_15 (Supplementary Table S5). However, the population of H14 O – H491 Nε2 Hε2 hydrogen bond was lower for h12_15 peptide.

**Figure 5:**
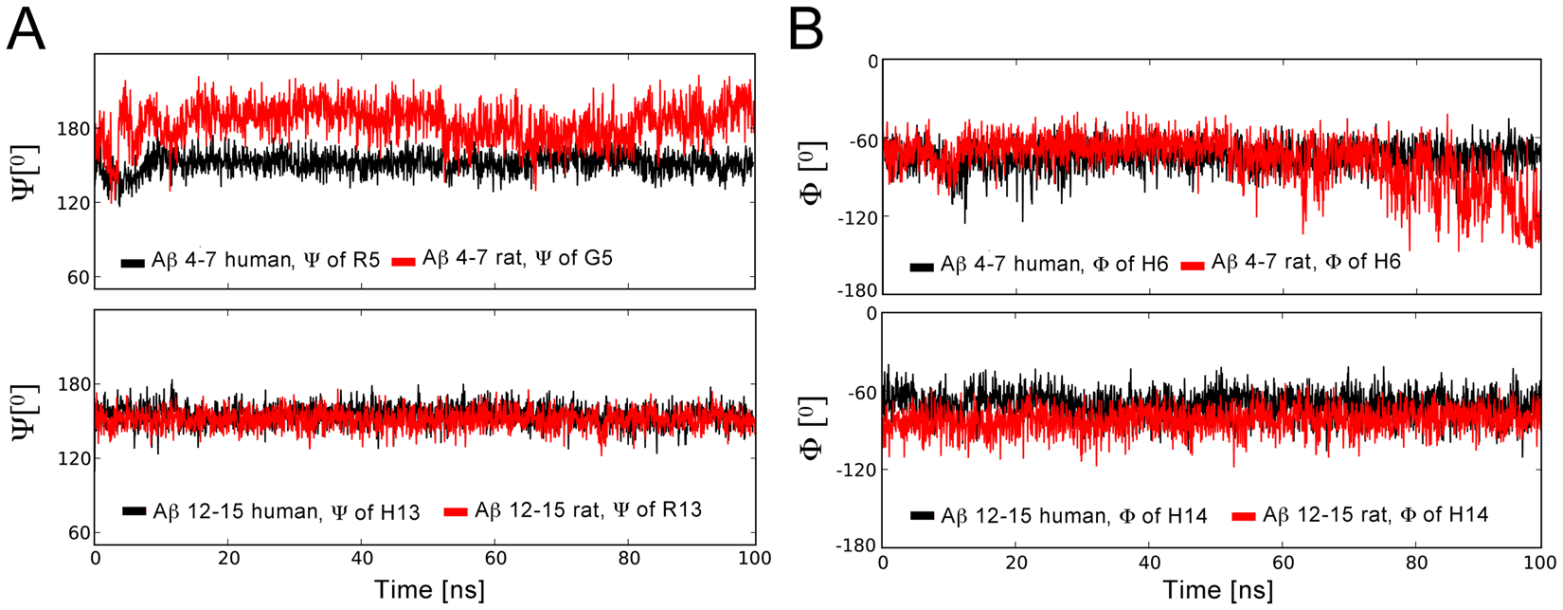
Motility of the backbone tetrahedral angles at scissile bond region for the Aβ tetrapeptides bound at N-ACE active site along the molecular dynamic trajectories: fluctuations of PSI angle of the residue, preceding peptide bond that is supposed to be hydrolyzed (**A**), fluctuations of PHI angle of the residue, next to the peptide bond that is supposed to be hydrolyzed (**B**).

The R5G substitution significantly changes the conformational behaviour of the tetrapeptide r4_7 (Fig. 3) and results in the increased backbone motility in the region of the scissile bond (Fig. 5). The characteristic peptide stabilization by hydrogen bonding with main chain atoms of A332 and A334 and side chains of H331, H491 and Y501 of N-ACE breaks down along the coarse of simulation (Fig. 4D, Supplementary Table S5). The peptide adopts a distorted extended conformation, where the position of the peptide bond between G5 and H6 residues moves along the N-ACE tunnel towards the C-terminus. The shift of the peptide position in the catalytic center is reflected by the formation of two new polar contacts: 1) a statistically significant hydrogen bond between the side chain of H331 and the backbone carbonyl oxygen of F4 instead of H6, and 2) a hydrogen bond between the hydroxyl group of Y501 and the backbone carbonyl oxygen of the N-terminal capping group instead of the backbone carbonyl oxygen of G5, which is observed by the end of the trajectory (Fig. 4D). Thus, the R5G substitution destabilizes the Michaelis complex of ratAβ fragment 4–7 with N-ACE. This explains the absence of endopeptidase activity toward the G5-H6 peptide bond of ratАβ(1-16).

The stability of the modelled complex between N-ACE and r12_15 correlates with the observed hydrolysis of bond Arg13-His14 in ratAβ(1-16). However, the Аβ fragment 12VHHQ15 which is not hydrolyzed by N-ACE also forms a well-stabilized complex in the active site of the enzyme as well as ratАβ fragment 12VRHQ15. The lack of hydrolysis of C-amidated Аβ(1-16) species by N-ACE can be explained by the influence of the Y10F substitution on the process. Indeed, the scissile bond of angiotensin I links phenylalanine and histidine residues, meaning that the P1 position in the N-ACE active site is well suited for bulky aromatic side chains, like the imidazole ring. The Y10F substitution appears as the fourth residue toward the N-terminus from the scissile bond R13-H14 of 12VRHQ15. The substrate tunnel of N-ACE forces an extended conformation on the ligand peptide, where each residue occupies a distinct pocket^53^. The interactions within these pockets govern substrate specificity of the enzyme^53^. Thus, the replacement of phenylalanine 10 by tyrosine which carries a hydroxyl group on the side-chain benzene ring can result in the destabilization of the position of the 12VHHQ15 substrate in the pocket and can lead to the loss of the catalytic activity toward the H13-H14 peptide bond of Аβ(1-16). In line with these considerations, several known peptide substrates of N-ACE have residues with significantly different from tyrosine shapes of side chains at fourth position toward N-terminus from the scissile bond (Supplementary Table S4).

The switch from the usual for ACE carboxypeptidase activity to the endoproteolytic one may be due to the specificity of the Аβ structure. As it was shown in this study, the endoproteolytic activity was observed only for peptides with a blocked carboxylic group at the C-end, i.e. for those without a negative charge in this crucial for ACE recognition region, and at the same time, the first amino acid in the Аβ peptide is an aspartic acid which carries a free carboxylic group (moreover, another negatively charged carboxylic amino acid, glutamate, is found in the third position). Thus in case of Аβ, the negative charge at its C-end is absent, but instead a negative charge is present on the N-end of Aβ(1-16)-[Amide]. This leads to an error in the recognition mechanism of ACE, and the enzyme instead of the C-terminal carboxylic group binds to the side chain group of Asp1 at the N-terminus. This error probably occurs at the entrance to the channel of the active site of the N-domain, where notable differences in hydrophobicity and charge are observed in the lid-like structure comprising of helices α1, α2 and α3^56^.

The probability of this assumption is also confirmed by the structure of the unique natural substrate of ACE toward which endoproteolitic activity of ACE was demonstrated, a regulatory peptide, luliberin, (gonadotropin releasing hormone, GnRH or LHRH)^57^, from which the N-domain of ACE cleaves an N-terminal tripeptide. This hormone is synthesized in the organism with a modified C-terminal amino-acid residue (Pyr-HWSYGLRPG-[Amide]) and a negatively charged pyroglutamate residue at the N-terminus. This unusual structure of luliberin with a blocked C-terminal carboxylate and a negatively charged N-terminus is similar to that of Aβ(1-16)-[Amide]. Thus, a common binding mechanism for both of these substrates by N-ACE, in which the N-terminus of the peptide imitates the C-end of a typical ACE substrate, can be assumed. In regard with this result, it is interesting to search for new ACE substrates, towards which the enzyme could also demonstrate its endoproteolytic action, among peptides and proteins whose sequence begins with negatively charged amino acid residues like aspartate and glutamate.

### Hypothesis: N-ACE aggravates the course of AD through generating isoAβ(6-x) species

Observational studies indicate that increased activity of ACE^21–23,58^, as well as inhibition of interactions between ACE and Аβ^33–37^, appear to be important for modulating AD, but the molecular mechanism of action of ACE on the development of the disease remains unknown. In the current study, we have shown that C-amidated peptides corresponding to the metal binding domains of human and rat Аβs are efficiently cleaved at the Arg-His bonds (Arg5-His6 and Arg13-His14, respectively) by the N-domain of ACE, which acts as an arginine specific endopeptidase. Our data also shows that C-terminal amidation is necessary and sufficient for such N-ACE action on these Aβ species. Molecular modelling has demonstrated that these Aβ substrates enter the active site of N-ACE with their N-termini. Since the N-terminal residues 1-16 form an independent folding unit in the full-length Aβ^45,48,59–61^, one can rationally suggest that N-ACE cleaves the same bond not only in Aβ(1-16)-[Amide] and [Acety]-Aβ(1-16)- [Amide], but also in physiologically significant longer Aβ species, including Aβ(1-40) and Aβ(1-42). In contrast to N-ACE, C-ACE demonstrates the usual for ACE carboxypeptidase activity for all non-amidated human Aβ peptides under study and for [Acetyl]-ratAβ(1-16)-[Amide].

Rats and mice are invulnerable to AD-like pathologies^49,50^, but for human beings and all other mammalians which suffer of AD, limited hydrolysis of Aβ by N-ACE resulting in the formation of Аβ(6-x) species may have dangerous consequences. Structurally modified Aβ molecules initiate AD-linked amyloidogenesis of endogenous Aβ in animal models^6^ probably through the aggregation seed mechanism^62^. One of such potential seeding agents is supposed to be Aβ carrying the isomerized Asp7 residue (isoAβ)^63,64^. IsoAβ appears to be involved in the AD pathogenesis by means of its zinc-dependent interactions with endogenous Aβ resulting in the formation of zinc-bound heterodimeric seeds causing Aβ aggregation^65^.

Results from our recent study suggest that removal of the N-terminal region 1-5 from Aβ and isoAβ enhances the ability of respective N-truncated Aβ(6-x) and isoAβ(6-x) species to form zinc-mediated oligomers^66^. It is worth noting that isoAβ is cleaved by N-ACE much more efficiently than native Aβ^32^, and at the same time isoAβ(6-x) is immensely more susceptible to zinc-driven oligomerization^66^. Thus, inhibitors of ACE should mainly suppress the formation of isoAβ(6-x) species, what could explain the positive effect of these inhibitors on patients with AD^33–37^ and the slowing of neurodegeneration in animal AD models^38–40^.

Translating the role of isoAβ as a trigger of amyloidogenesis in AD animal models^63,64^ for human patients and taking into account above mentioned considerations, we have assumed the following scenario of N-ACE linkage to AD: (i) in a healthy organism endoproteolytical cleavage of native Aβ at the Arg5-His6 bond is quite rare and a rather normal processing event; (ii) when isoAβ species are formed (for example, due to Aβ ageing, neurotrauma, etc), a rapid limited hydrolysis of these species by N-ACE results in the formation of isoAβ(6-x) molecules which are extremely susceptible to zinc-induced oligomerization and by this reason should significantly enhance the pathological aggregation of endogenous Aβ. This scenario, on one hand, supports the amyloid cascade hypothesis of AD, and, on the other hand, for the first time links together several molecular agents such as Aβ, isoAβ, zinc ions, and ACE, in a potentially pathogenic network.

In summary, the presented study showed that N-ACE specifically cleaves synthetic C-amidated peptide analogs of the metal-binding domains of Aβ and ratAβ at Arg-His bonds 5-6 and 13-14, respectively. Computer modeling provided evidence that these peptides enter the active site of N-ACE with their N-termini, thus assuming that full-length Aβ and ratAβ molecules should be hydrolyzed by ACE in the same way as the C-amidated peptides under the study. Concerning the possible clinical applications, our results indicate that N-ACE seems to play an aggravating role in AD pathogenesis by generating extremely susceptible to zinc-induced oligomerization isoAβ(6-x) species, and thus N-ACE inhibitors should slow down AD progression.

## Methods

### Reagents

H_2_^18^O with 95–98% ^18^O content was purchased from Cambridge Isotope Laboratories (Andover, MA, USA), α-cyano-4-hydroxycinnamic acid (HCCA) was from Bruker Daltonics (Bremen, Germany). Trypsin was purchased from Promega (Madison, WI, USA). All other reagents were of analytical grade or better and were obtained from Sigma-Aldrich (St. Louis, MO, USA) unless otherwise stated.

### Amyloid-β peptides

Synthetic peptides (purity > 95% checked by reversed-phase high-performance liquid chromatography) Asp-Ala-Glu-Phe-Arg^5^-His-Asp- Ser-Gly-Tyr^10^-Glu-Val-His-His-Gln^15^-Lys (Aβ(1-16)), Asp-Ala-Glu-Phe-Arg^5^-His-Asp- Ser-Gly-Tyr^10^-Glu-Val-His-His-Gln^15^-Lys-[NH_2_] (Aβ(1-16)-[Amide]), [CH_3_CO]-Asp-Ala-Glu-Phe-Arg^5^-His-Asp- Ser-Gly-Tyr^10^-Glu-Val-His-His-Gln^15^-Lys-[NH_2_] ([Acetyl]-Aβ(1-16)-[Amide]), and [CH_3_CO]-Asp-Ala-Glu-Phe-Gly^5^-His-Asp- Ser-Gly- Phe^10^-Glu-Val-Arg-His-Gln^15^-Lys-[NH_2_] ([Acetyl]-ratAβ(1-16)-[Amide]) were purchased from Sigma-Genosys (The Woodlands, TX, USA). Purity and sequence of the peptides under study were confirmed by accurate mass-measurement and MS/MS fragmentation using an LTQ FT Ultra tandem mass-spectrometer (Thermo Finnigan, Germany) as described previously^67^. MALDI TOF mass spectra (see the section 3.5. for experiment details) of the peptides incubated for 130 min in 50 mM sodium bicarbonate buffer (pH 7.8) or 25 mM barbital buffer (pH 7.4) (not shown) have demonstrated that the peptides in both buffer systems: (i) are homogenous; (ii) do not contain neither significant contaminants, nor degradation products; and (iii) do not undergo spontaneous degradation during 130 min of aging.

### Angiotensin –converting enzyme (ACE)

The N-domain and C-domains of bovine ACE (N-ACE and C-ACE), homogenous according to sodium dodecyl sulfate polyacrylamide gel electrophoresis (SDS-PAGE), were provided by Dr P.V. Binevski (Moscow State University, Russia). Enzymatic activities of the ACE domains were measured by a fluorometric method using the Z-Phe-His-Leu substrate as described previously^32^. Briefly, 2 mL of the reaction mixture contained barbital buffer (25 mM, pH 7.4), NaCl (50 mM for N-ACE assay or 200 mM for C-ACE assay), ZnCl_2_ (1 mM), N-ACE (0.02 mM) or C-ACE (0.02 mM) and Z-Phe- His-Leu (50 mM), which was added to initiate the reaction. The mixture was incubated at 37 °C for 30 min. Lisinopril (10 mM) was added 20 min before substrate addition. The reaction was terminated by adding 0.4 mL of 2 N NaOH. Samples were processed by adding 1 mL of bidistillate water, 0.1 mL of 1% o-phthaldialdehyde and after 6 min 0.2 mL of 6 N HCl. Fluorescence was measured at an excitation wavelength of 370 nm and at emission wavelength of 500 nm. Fluorescence of a standard solution of His-Leu (10 nM) was measured in duplicate, simultaneously with that of the samples and blanks. The N- and C-domain activities were 27.6 nM and 17.1 nM His-Leu/min/mg, respectively.

### Enzymatic digestion

The hydrolysis of Aβ(1-16), Aβ(1-16)-[Amide] or [Acetyl]-Aβ(1-16)-[Amide] by the N- or C-ACE domains was performed for 10–40 min at 37 °C in 25 μL of the reaction mixture containing 40 μM of the respective peptide, 0.02 μM N-ACE or 0.02 μM C-ACE, 50/200 mM NaCl (for N- or C-domain, respectively), 1 μM ZnCl_2_, 50 mM sodium bicarbonate buffer (pH 7.8) or 25 mM barbital buffer (pH 7.4). The hydrolysis of [Acetyl]-ratAβ(1-16)-[Amide] by the N- or C-ACE domains was performed for 10-60 min at 37 °C in 23 μL of the reaction mixture containing 20 μM of the peptide, 0.2 μM N-ACE or 0.2 μM C-ACE, 50/200 mM NaCl (for N- or C-domain, respectively), 1 μM ZnCl_2_, 50 mM sodium bicarbonate buffer (pH 7.8) or 25 mM barbital buffer (pH 7.4). For MS analysis, the digestion process was terminated by adding a 5 μL aliquot of each reaction mixture to 15 μL of 0.5% trifluoroacetic acid (TFA) to obtain an acidic solution (final pH~3); then 0.5 μL of this solution was used to prepare the MALDI probe as described in the section 2.5.

### Mass spectrometry (MS)

Due to the low complexity of the studied system – only one highly purified peptide-substrate and enzyme per sample – high mass-accuracy and MS/MS confirmation were not necessary for reliable identification of the reaction products, while for quantitative measurements fast sample analysis procedure and low sample and H_2_O^18^ consumption were required, thus it was decided to use Bruker Microflex MALDI TOF instrument (Bruker Daltonics, Germany) for the study. Mass spectra were acquired in a positive-ion reflector mode, 200–500 laser shots were summed per spectrum. To prepare the matrix solution, HCCA was dissolved to a concentration of 10 mg/mL in acetonitrile /0.1% TFA (70:30 v/v). Usually, for MALDI probe preparation, the dried-droplet method was used: 0.5 μL of 2% TFA was mixed with 0.5 μL of the sample (0.5–2 pmol per target) and 0.5 μL of the matrix solution, then loaded onto a MALDI sample plate and measured by MS.

### Quantitative determination of ACE digestion products using ^18^O-labeled internal standards

A method for quantitating the products of enzyme degradation has been based on the use of MALDI- TOF MS with internal ^18^O-labeled standards. A simple procedure allows to produce such internal standards for the tested sample by enzymatic hydrolysis of the same sample (of a known concentration) in ^18^O-water as described earlier^68^. Briefly, to prepare the ^18^O-labeled internal standards, hydrolysis was performed at 37 °C in 25 µL of ^18^O-water solution containing 20 µM of an appropriate peptide, 50 mM of ammonium bicarbonate (pH 7.8), and 1 µg of trypsin. In order to completely hydrolyze the substrate the reaction was incubated for 48 h, and then the sample was kept at −20 °C until analysis. To obtain the final standard solution, 5 µL of the terminated reaction mixture were added to 45 µL of the matrix solution (see previous section). For quantitation assay, 5 µL of the final standard solution were mixed with an equal volume of an ACE digestion mixture pre-incubated for 10, 20, 40 and 60 min, then, 1 μL of the resulting mixture was applied directly onto the MALDI target plate and subjected to MALDI-TOF MS analysis to obtain the isotopic pattern of the corresponding analyte/internal standard mixture. The previously described algorithm^68^ was used to calculate the absolute concentration of the peptide of interest on the basis of experimentally determined isotopic patterns of the analyte and the ^18^O-labeled standard (of a known concentration) and of the analyte/internal standard mixture. The method error was estimated to be less than 10%^51^.

### Molecular modelling studies

#### Modelling of Michaelis complexes of Aβ peptides with N-ACE and force-field parameterization

The models of the Michaelis complex have been constructed for the N-domain of ACE, bound with four tetrapeptide fragments of Aβ (4FRHD7 and 12VHHQ15) and ratAβ (4FGHD7 and 12VRHQ15). All tetrapeptides were acetylated at the N-terminus and amidated at the C-terminus. The models have been build using the crystallographic structure of N-domain of somatic ACE with lisinopril, zinc ion bound in the active site and chlorine ion at Y202/R500 site (PDB code 2C6N). The tetrapeptides have been fitted in the active site tunnel by manual superimposition of the main chain peptide atoms on the corresponding atoms of lisinopril^54^. The lisinopril zinc-coordinating carboxyl group has been replaced by a water molecule. The fitted peptide chains of the obtained models have been minimized using 100 steps of conjugate gradient minimization (Supplementary Fig. S6). Modelling has been accomplished using the Chimera software^69^.

The bonded plus electrostatic model has been used to describe zinc chelation^70^. Following the previously published studies of the ACE catalytic mechanism^54,55^, we have assumed a tetrahedral coordination of the zinc ion by the side chains of residues H361, H365, E389 and a water molecule (Supplementary Fig. S7). The force-field parameters for the zinc-chelating environment have been derived using ab-initio calculations in Gaussian 09w^71^. The local geometry of the zinc-binding interface has been optimized and force constants and atomic partial charges have been derived following the procedure implemented in the Metal Center Parameter Builder (MCPB) package^72^. The quantum mechanical calculations have been performed at the B3LYP level of theory with the 6–31 G* basis set. The force-field constants have been derived from the Cartesian Hessian matrix by the Seminario method^73^ and partial charges have been obtained from the Merz-Singh-Kollman charges using Restrained Electrostatic Potential (RESP) fitting^74^. Calculated force-field parameters are summerized in Supplementary Tables S1 and S2.

#### Molecular dynamics simulations

The molecular dynamics simulations have been performed using the GROMACS 4.6.5 software package^75^ and Amber ff99SB-ILDN force field^76^. The model of N-ACE complexed with a tetrapeptide has been placed in a cubic cell with a minimum distance between the protein and the box of 0.8 nm and solvated using TIP3P water molecules^77^. The total charge has been neutralized by Na^+^ ions. The chlorine ion at the Y202/R500 site of N-ACE was retained. The system was minimized using the steepest descent minimization algorithm. Positions for the protein complex atoms were restrained and the system was equilibrated with 100 ps of constant volume molecular dynamics followed by 100 ps of constant pressure molecular dynamic. The production of 0.1 µs molecular dynamics trajectory has been obtained. Calculations have been done with 2 fs integration steps at a constant pressure of 1 atm and temperature of 300 K using the Berendsen barostat and the velocity rescale method for the thermostat. The particle-mesh Ewald method^78^ has been implemented to treat long-range electrostatic interactions and the LINCS algorithm controlled the lengths of covalent bonds^79^. The procedure has been repeated for each of the four modelled complexes. Hydrogen bond population analysis has been done using h-bond utility of GROMACS 4.6.5^75^ and in-house written scripts.

Molecular dynamics calculations have been performed using the equipment of the shared research facilities of HPC computing resources at Lomonosov Moscow State University. Structure visualization has been done in PyMOL (Schrödinger, LLC).

### Data Availability Statement

The datasets generated during and/or analyzed during the current study are available from the corresponding author on reasonable request.

## Electronic supplementary material

Supplementary Information
