# Supplementary material for: N-domain of angiotensin-converting enzyme hydrolyzes human and rat amyloid-β(1-16) peptides as arginine specific endopeptidase potentially enhancing risk of Alzheimer’s disease
Source: Sci Rep. 2018 Jan 10;8:298. doi: 10.1038/s41598-017-18567-5 (PMC5762728; doi:10.1038/s41598-017-18567-5)
Supplement: Supplementary file 1 — Supplementary Information [file 41598_2017_18567_MOESM1_ESM.doc]

**SUPPLEMENTARY INFORMATION**

**N-domain of angiotensin-converting enzyme hydrolyzes human and rat amyloid-β(1-16) peptides as arginine specific endopeptidase potentially enhancing risk of Alzheimer’s disease**

Elena V. Kugaevskaya ^a^,♦ , Alexander V. Veselovsky ^a^,♦ , Maria I. Indeykina ^b,c,d^, Nina I. Solovyeva ^a^, Maria S. Zharkova ^a^, Igor A. Popov ^b,c,d^, Eugene N. Nikolaev ^b,c^, Alexey B. Mantsyzov ^e^, Alexander A. Makarov ^d^, Sergey A. Kozin ^d^,*

^a^ Orekhovich Institute of Biomedical Chemistry, Moscow, Russia

^b^ Emanuel Institute of Biochemical Physics of the Russian Academy of Sciences, Moscow, Russia

^c^ Moscow Institute of Physics and Technology, Dolgoprudnyi, Moscow Region, Russia.

^d^ Engelhardt Institute of Molecular Biology of the Russian Academy of Sciences, Moscow, Russia

^e^ Faculty of Fundamental Medicine, Lomonosov Moscow State University, Moscow, Russia

♦These authors contributed equally to the work

*Corresponding author at: Engelhardt Institute of Molecular Biology of the Russian Academy of Sciences, Vavilov str 32, Moscow 119991, Russia

Phone: +7 926 386 7020

Fax: +7 499 135 14 05

E-mail address: [kozinsa@gmail.com](mailto:kozinsa@gmail.com) (Sergey A. Kozin)

**Figures and Tables**

**Figure S1.** MALDI-TOF mass spectra of peptide substrates: (A) Aβ(1-16) (m/z 1954.8); (B) Aβ(1-16)-[Amide] (m/z 1953.9); (C) [Acetyl]-Aβ(1-16)-[Amide] (m/z 1995.8); (D) [Acetyl]-ratAβ(1-16)-[Amide] (m/z 1899.9).

**Figure S2.** MALDI-TOF mass spectra of reaction products obtained from N-ACE action on the following substrates: Aβ(1-16) (m/z 1954.8) (A), Aβ(1-16)-[Amide] (m/z 1953.9) (B), [Acetyl]-Aβ(1-16)-[Amide] (m/z 1995.8) (C), [Acetyl]-Aβ(1-16)-[Amide] (m/z 1995.8) and lisinopril (D). The observed products are Аβ(1-14) (m/z value 1698.6) (A), Aβ(6-16)-[Amide] (m/z 1335.6) (B,C). and [Acetyl]-Aβ(1-5) (m/z 679.3 – not shown) (C).

**Figure S3.** MALDI-TOF mass spectra of reaction products obtained from C-ACE action on the following substrates: Aβ(1-16) (m/z 1954.8) (A), Aβ(1-16)-[Amide] (m/z 1953.9) (B), [Acetyl]-Aβ(1-16)-[Amide] (m/z 1995.8) (C), Aβ(1-16) (m/z 1954.8) and lisinopril (D). The observed products are Аβ(1-13) (m/z value 1561.5) (A) and Aβ(1-14) (m/z 1698.6) (A).

**Figure S4.** MALDI-TOF mass spectra of reaction products obtained from the incubation for 60 min of [Acetyl]-ratAβ(1-16)-[Amide] (m/z 1899.9) with N-ACE (A), with N-ACE in the presence of lisinopril (B), with C-ACE (C), and with C-ACE in the presence of lisinopril (D). The observed products are [Acetyl]-Аβ(1-13) (m/z 1507.5) (A,C), [Acetyl]-ratАβ(1-14) (m/z 1644.9) (C), [Acetyl]-ratАβ(1-15) (m/z 1772.9) (C).

**Figure S5.** Zoom of MALDI-TOF mass spectra presenting isotopic patterns of the major MS signals obtained from the following peptides: unlabeled [Acetyl]-ratAβ(1-13) (m/z 1507.5) (A); ^18^O-[Acetyl]-ratAβ(1-13) (B); ^18^O-[Acetyl]-ratAβ(1-13) added to the reaction mixture, containing N-ACE and [Acetyl]-ratAβ(1-16) (C); ^18^O-[Acetyl]-ratAβ(1-13) added to the reaction mixture, containing C-ACE and [Acetyl]-ratAβ(1-16) (D). Incorporation of 1 and 2 ^18^O labels is observed.

**Figure S6.** Superimposition of the model of h4_7 tetrapeptide (shown in purple) and the experimental lisinopril structure (shown in green) bound in N-ACE active site. The water molecule, which completes tetrahedral zinc coordination sphere in the model of h4_7 / N-ACE Michaelis complex, is shown by sticks. The model was used as the starting structure for the molecular dynamic simulation of h4_7 / N-ACE complex.

**
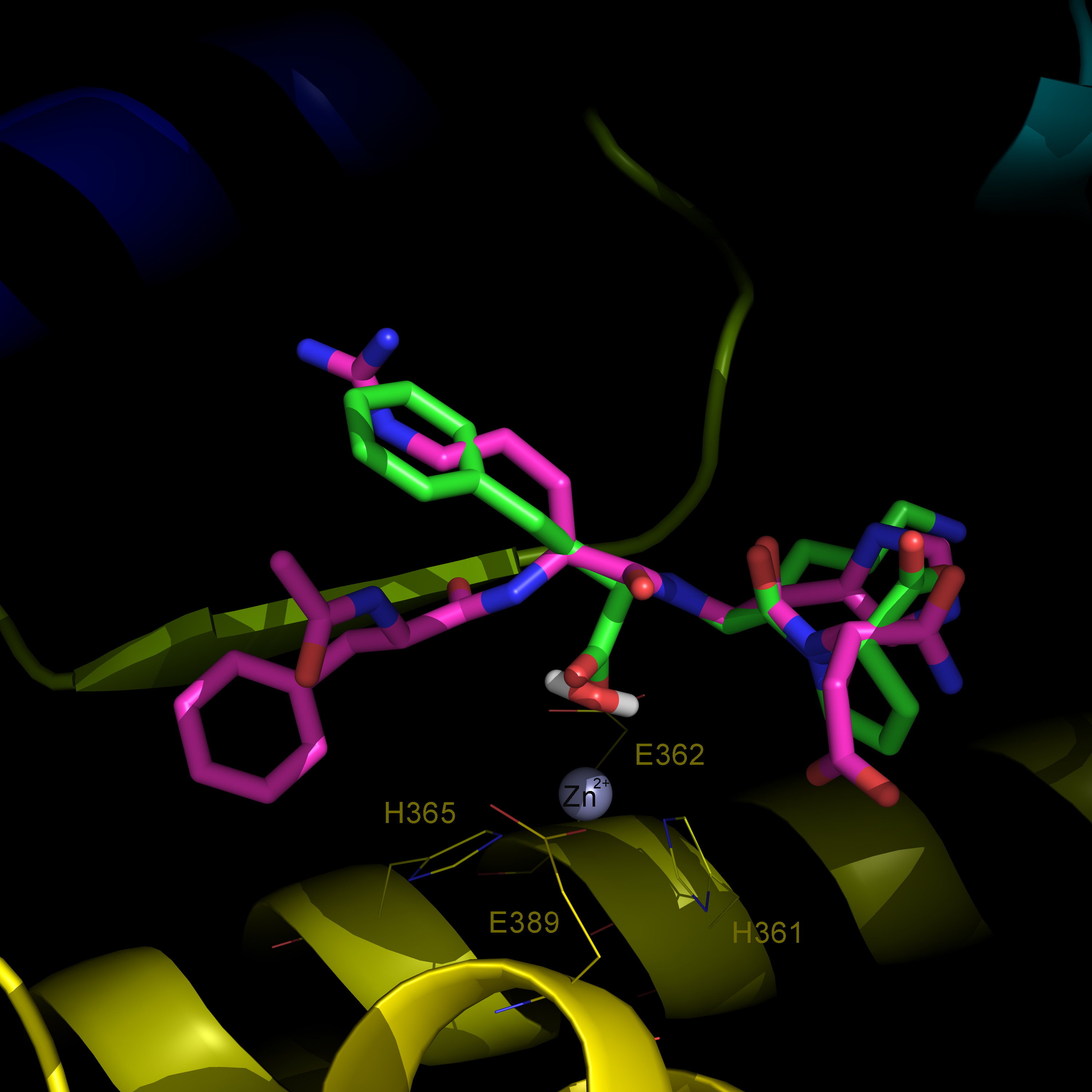
**

**Figure S7.** Zinc coordination sphere after DFT minimization.

**
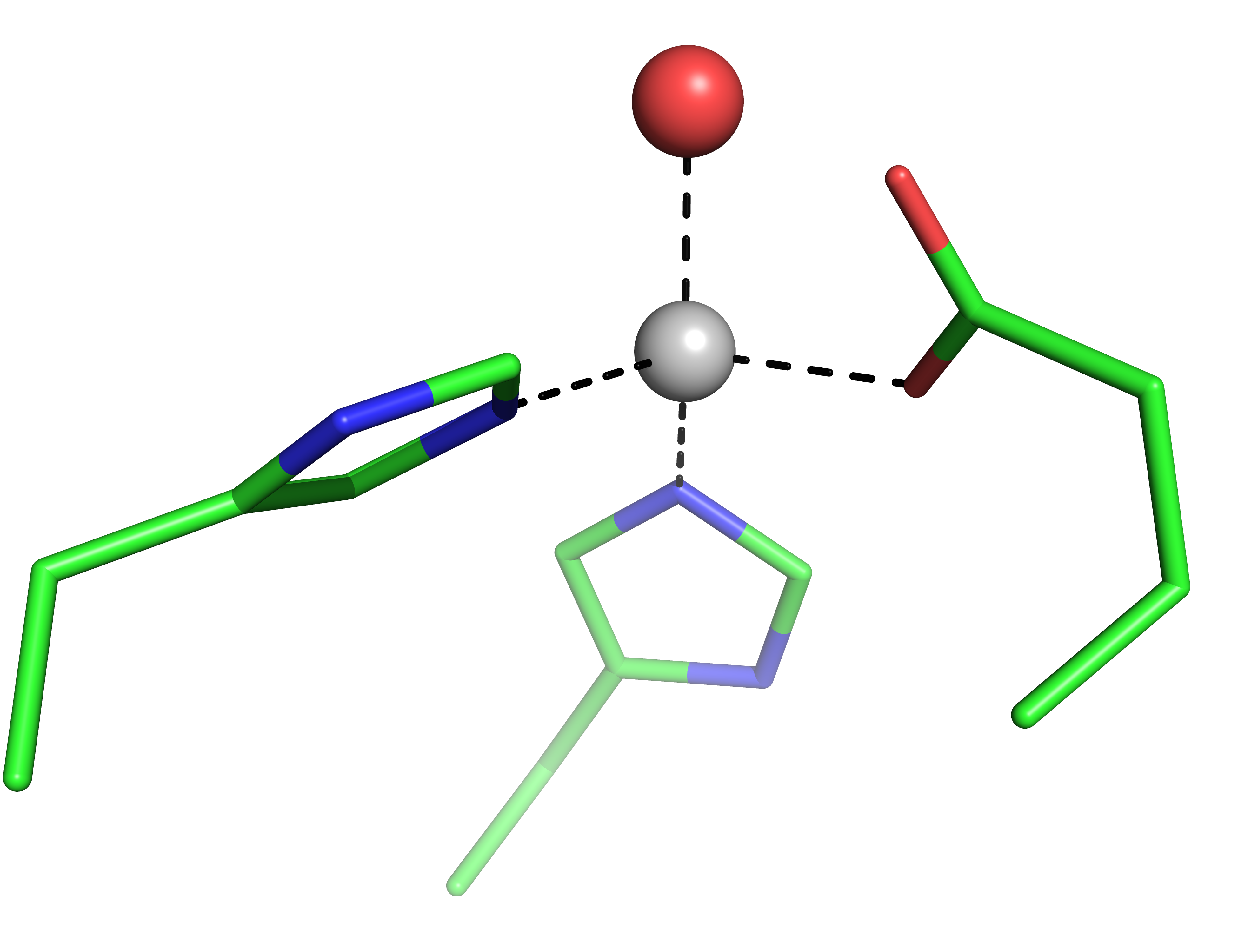
**

**Figure S8.** Backbone heavy atoms RMSD fluctuation for N-ACE complexes along 100 ns molecular dynamic trajectories.


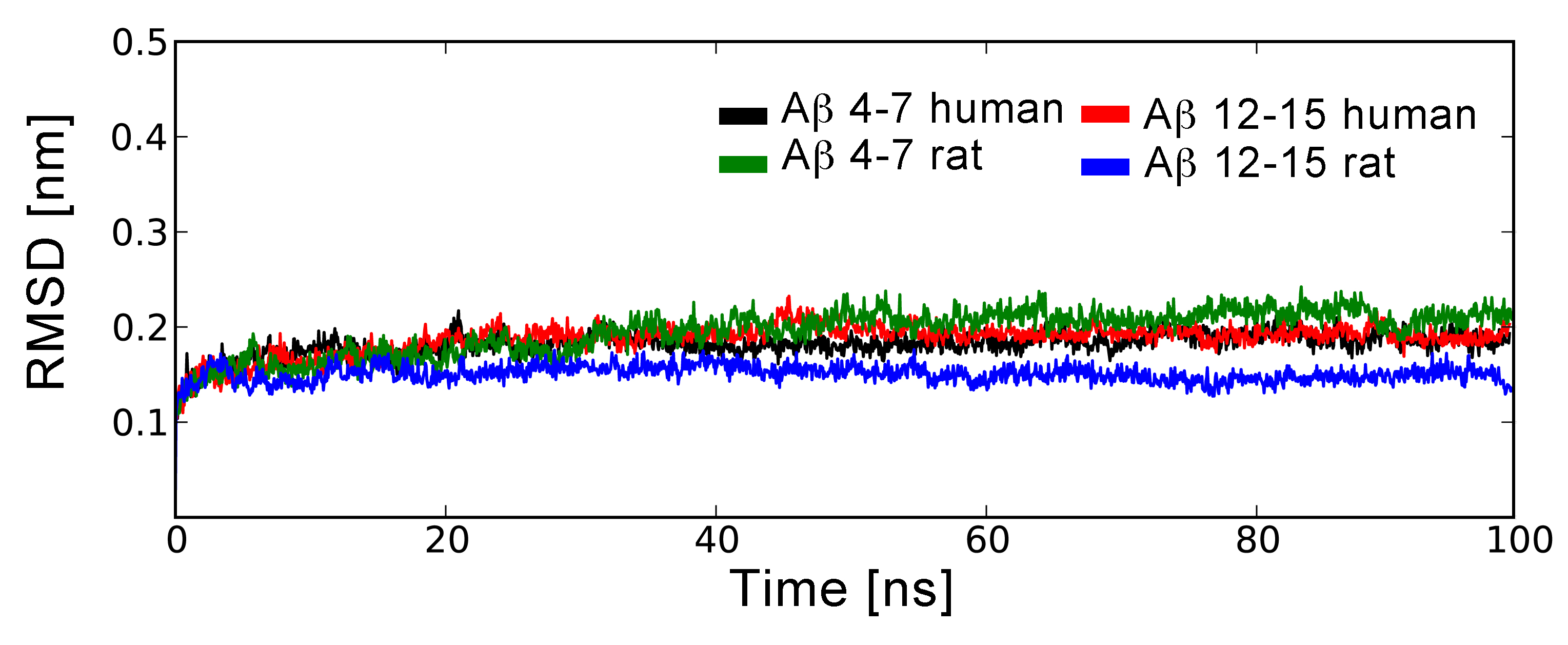


**Table S1.** Partial charges used for the modeling of zinc-binding center. Peptide atom names correspond to that in Amber ff99SB-ILDN.

| **Residue** | **Atom name** | **Charge** |
| --- | --- | --- |
| H361 | N | -0.415700 |
|  | CA | 0.018800 |
|  | C | 0.597301 |
|  | O | -0.567901 |
|  | CB | -0.168803 |
|  | CG | 0.022420 |
|  | CD2 | -0.016229 |
|  | ND1 | -0.165402 |
|  | CE1 | -0.031309 |
|  | NE2 | -0.233391 |
|  | H | 0.335978 |
|  | HA | 0.134359 |
|  | HB2 | 0.083726 |
|  | HB3 | 0.083726 |
|  | HD1 | 0.358277 |
|  | HD2 | 0.128383 |
|  | HE1 | 0.192335 |
|  |  |  |
| H365 | N | -0.415700 |
|  | CA | 0.018800 |
|  | C | 0.597301 |
|  | O | -0.567901 |
|  | CB | -0.099285 |
|  | CG | 0.041639 |
|  | CD2 | -0.047168 |
|  | ND1 | -0.096176 |
|  | CE1 | -0.058681 |
|  | NE2 | -0.256384 |
|  | H | 0.372747 |
|  | HA | 0.072299 |
|  | HB2 | 0.019808 |
|  | HB3 | 0.019808 |
|  | HD1 | 0.334944 |
|  | HD2 | 0.141922 |
|  | HE1 | 0.170922 |
|  |  |  |
| E389 | N | -0.516301 |
|  | CA | 0.039700 |
|  | C | 0.536601 |
|  | O | -0.581901 |
|  | CB | 0.255419 |
|  | CG | -0.339974 |
|  | CD | 0.713278 |
|  | OE1 | -0.663222 |
|  | OE2 | -0.582327 |
|  | H | 0.353002 |
|  | HA | 0.109739 |
|  | HB2 | -0.016640 |
|  | HB3 | -0.016640 |
|  | HG2 | 0.081610 |
|  | HG3 | 0.081610 |
|  |  |  |
| H_2_O | O | -0.748925 |
|  | H1 | 0.442445 |
|  | H2 | 0.473406 |
|  |  |  |
| Zinc ion | ZN | 0.773654 |
|  |  |  |

**Table S2.** Force constants, used for the modeling of zinc-binding center.

| **Bond** | **Force constant**  **[kcal/(mol*Å^2^)]** | **Bond length**  **[Å]** |
| --- | --- | --- |
| Zn – H361_Ne2 | 89.4 | 1.9841 |
| Zn – H365_Ne2 | 85.6 | 1.9936 |
| Zn – E389_Oe1 | 88.4 | 1.9378 |
| Zn – H2O_O | 59.4 | 2.0363 |
|  |  |  |
| **Angle** | **Force constant**  **[kcal/(mol*rad^2^)]** | **Angle**  **[^o^]** |
| E389_Cd – E389_Oe1 - Zn | 43.54 | 114.01 |
| H361_Ce1 – H361_Ne2 - Zn | 65.57 | 116.80 |
| H365_Ce1 – H365_Ne2 - Zn | 47.29 | 123.16 |
| H361_Cd2 – H361_Ne2 - Zn | 54.97 | 133.15 |
| H365_Cd2 – H365_Ne2 - Zn | 47.92 | 130.24 |
| H361_Ne2 – Zn – H365_Ne2 | 42.39 | 112.26 |
| H361_Ne2 – Zn – E389_Oe1 | 40.34 | 97.61 |
| H361_Ne2 – Zn – H2O _O | 38.52 | 130.49 |
| H365_Ne2 – Zn – E389_Oe1 | 35.97 | 118.06 |
| H365_Ne2 – Zn – H2O_O | 37.22 | 101.60 |
| E389_Oe1 – Zn – H2O_O | 41.19 | 97.00 |
|  |  |  |

**Table S3.** Average over the trajectory angles for zinc coordination sphere.

| **Angle definition** | **Angle value [^o^]** |
| --- | --- |
| *Aβ Human 4-7* |  |
| H361_Ne2 – Zn – H365_Ne2 | 108.79 ± 3.96 |
| H361_Ne2 – Zn – E389_Oe1 | 90.41 ± 3.99 |
| H361_Ne2 – Zn – H2O _O | 133.35 ± 4.10 |
| H365_Ne2 – Zn – E389_Oe1 | 115.46 ± 4.58 |
| H365_Ne2 – Zn – H2O_O | 109.30 ± 4.51 |
| E389_Oe1 – Zn – H2O_O | 95.87 ± 4.41 |
| *Aβ Human 12-17* |  |
| H361_Ne2 – Zn – H365_Ne2 | 110.51 ± 4.03 |
| H361_Ne2 – Zn – E389_Oe1 | 89.07 ± 3.93 |
| H361_Ne2 – Zn – H2O _O | 133.88 ± 4.26 |
| H365_Ne2 – Zn – E389_Oe1 | 117.57 ± 4.57 |
| H365_Ne2 – Zn – H2O_O | 107.41 ± 4.12 |
| E389_Oe1 – Zn – H2O_O | 95.17 ± 4.57 |
| *Aβ Rat 4-7* |  |
| H361_Ne2 – Zn – H365_Ne2 | 110.48 ± 3.88 |
| H361_Ne2 – Zn – E389_Oe1 | 90.51 ± 3.61 |
| H361_Ne2 – Zn – H2O _O | 133.93 ± 4.13 |
| H365_Ne2 – Zn – E389_Oe1 | 116.75 ± 4.42 |
| H365_Ne2 – Zn – H2O_O | 107.62 ± 4.23 |
| E389_Oe1 – Zn – H2O_O | 94.05 ± 4.38 |
| *Aβ Rat 12-17* |  |
| H361_Ne2 – Zn – H365_Ne2 | 111.56 ± 4.35 |
| H361_Ne2 – Zn – E389_Oe1 | 87.91 ± 4.46 |
| H361_Ne2 – Zn – H2O _O | 133.81 ± 4.25 |
| H365_Ne2 – Zn – E389_Oe1 | 116.92 ± 4.16 |
| H365_Ne2 – Zn – H2O_O | 107.17 ± 4.31 |
| E389_Oe1 – Zn – H2O_O | 95.37 ± 4.89 |

**Table S4.** Sequences of N-ACE substrates, aligned by residues, forming scissile bond (residues are underlined). The residues at fourth position toward N-terminus from the scissile bond are in blue. The last sequence represents human Aβ(1-16)aligned by HH fragment.

BK                RPPGFSPFR

Des-Arg9 BK RPPGFSPF

GnRH             pEHWSYGLRPnG

Ang I           DRVYIHPFHL

Ang 1-7 DRVYIHP

Heptapeptide YGGFMRF

Octapeptide YGGFMRGL

Substance P RPKPQQFFGLnM

Substance P RPKPQQFFGLM

analogue

Neurotensin ELYENKPRRPYIL

AB 16 rat DAEFGHDSGFEVRHQK

AB 16 hum DAEFRHDSGYEVHHQK

AB 16 hum DAEFRHDSG**Y**EV**HH**QK

*GnRH - gonadotropin-releasing hormone, Ang - angiotensin, BK – bradykinin, pE- N-terminus pyroglutamic acid, nG and nM – amidated C-terminal glycine and C-terminal methionine.

**Table S5.** Analysis of populations for the hydrogen bonds formed between Aβ tetrapeptides and N-ACE along the molecular dynamic trajectories. Statistics for hydrogen bonds with populations >10% is shown. All Aβ back-bone contacts within one line of the table correspond to the same hydrogen bonding receptor group. Key contacts for the scissile bond stabilization are bolded.

|  | **h4_7** | | | | | **h12_15** | | | | | | **r12_15** | | | | | | **r4_7** | | | | |
| --- | --- | --- | --- | --- | --- | --- | --- | --- | --- | --- | --- | --- | --- | --- | --- | --- | --- | --- | --- | --- | --- | --- |
| **N** | **h4_7**  **group** | | **N-ACE**  **group** | | **P**  **[%]** | **h12_15 group** | | **N-ACE**  **group** | | | **P**  **[%]** | **r12_15 group** | | **N-ACE**  **group** | | | **P**  **[%]** | **r4_7 group** | | **N-ACE**  **group** | | **P [%]** |
| ***A****β* ***peptide back-bone hydrogen bonds with N-ACE*** | | | | | | | | | | | | | | | | | | | | | | |
| 1 |  |  |  |  |  |  |  | |  |  |  |  |  | |  |  |  | F4 | O | H331 | NE2-HE2 | 30.4 |
| 2 | F4 | O | A334 | N-H | 50.5 | V12 | O | | A334 | N-H | 90.8 | V12 | O | | A334 | N-H | 90.4 |  |  |  |  |  |
| 3 | F4 | N-H | A334 | O | 53.7 | V12 | N-H | | A334 | O | 90.0 | V12 | N-H | | A334 | O | 74.6 |  |  |  |  |  |
| ***4*** | ***R5*** | ***O*** | ***Y501*** | ***OH-HH*** | ***96.5*** | ***H13*** | ***O*** | | ***Y501*** | ***OH-HH*** | ***57.5*** | ***R13*** | ***O*** | | ***Y501*** | ***OH-HH*** | ***89.2*** | ***G5*** | ***O*** | ***Y501*** | ***OH-HH*** | ***65.7*** |
| ***5*** | ***H6*** | ***N-H*** | ***A332*** | ***O*** | ***93.4*** | ***H14*** | ***N-H*** | | ***A332*** | ***O*** | ***97.9*** | ***H14*** | ***N-H*** | | ***A332*** | ***O*** | ***97.6*** | ***H6*** | ***N-H*** | ***A332*** | ***O*** | ***77.6*** |
| ***6*** | ***H6*** | ***O*** | ***H331*** | ***NE2-HE2*** | ***10.8*** | ***H14*** | ***O*** | | ***H331*** | ***NE2-HE2*** | ***71.2*** | ***H14*** | ***O*** | | ***H331*** | ***NE2-HE2*** | ***12.7*** | ***H6*** | ***O*** | ***H331*** | ***NE2-HE2*** | ***22.8*** |
| ***7*** | ***H6*** | ***O*** | ***H491*** | ***NE2-HE2*** | ***50.4*** | ***H14*** | ***O*** | | ***H491*** | ***NE2-HE2*** | ***19.9*** | ***H14*** | ***O*** | | ***H491*** | ***NE2-HE2*** | ***49.4*** | ***H6*** | ***O*** | ***H491*** | ***NE2-HE2*** | ***10.5*** |
| 8 | D7 | O | Q259 | NE2-HE21 | 17.8 | Q15 | O | | Q259 | NE2-HE21 | 10.7 |  |  | |  |  |  |  |  |  |  |  |
| 9 | D7 | O | K489 | NZ-HZ1 | 61.9 | Q15 | O | | K489 | NZ-HZ1 | 41.9 | Q15 | O | | K489 | NZ-HZ1 | 41.8 | D7 | O | K489 | NZ-HZ1 | 16.1 |
| 10 | D7 | O | Y498 | OH-HH | 80.2 | Q15 | O | | Y498 | OH-HH | 61.8 | Q15 | O | | Y498 | OH-HH | 64.3 | D7 | O | Y498 | OH-HH | 30.5 |

**Table S5.** (Continue) Analysis of populations for the hydrogen bonds formed between Aβ tetrapeptides and N-ACE along the molecular dynamic trajectories. Statistics for hydrogen bonds with populations >10% is shown.

|  | **h4_7** | | | | | **h12_15** | | | | | | **r12_15** | | | | | **r4_7** | | | | |
| --- | --- | --- | --- | --- | --- | --- | --- | --- | --- | --- | --- | --- | --- | --- | --- | --- | --- | --- | --- | --- | --- |
| **N** | **h4_7**  **group** | | **N-ACE**  **group** | | **P**  **[%]** | **h12_15 group** | | **N-ACE**  **group** | | | **P [%]** | **r12_15**  **group** | | **N-ACE**  **group** | | **P [%]** | **r4_7**  **group** | | **N-ACE**  **group** | | **P**  **[%]** |
| ***A****β* ***peptide side chain hydrogen bonds with N-ACE*** | | | | | | | | | | | | | | | | | | | | | |
| 11 | R5 | NH2-HH21 | D43 | OD2 | 87.6 | Q15 | OE1 | | Q259 | NE2-HE21 | 48.9 | R13 | NE-HE | H331 | ND1 | 20.6 | H6 | ND1-HD1 | A332 | O | 32.6 |
| 12 | R5 | NH1-HH11 | D43 | OD1 | 38.2 | Q15 | OE1 | | K489 | NZ-HZ1 | 11.0 | R13 | NE-HE | H331 | O | 39.6 | D7 | OD1 | Q259 | NE2-HE21 | 19.8 |
| 13 | R5 | NH1-HH11 | D43 | OD2 | 46.7 |  |  | |  |  |  | R13 | NH1-HH11 | D43 | OD1 | 11.0 | D7 | OD2 | Q259 | NE2-HE21 | 33.8 |
| 14 | R5 | NH1-HH11 | H331 | O | 52.8 |  |  | |  |  |  | R13 | NH1-HH11 | D43 | OD2 | 95.2 | D7 | OD2 | K489 | NZ-HZ1 | 48.4 |
| 15 | H6 | ND1-HD1 | A332 | O | 40.8 |  |  | |  |  |  | R13 | NH2-HH21 | D43 | OD1 | 85.6 | D7 | OD2 | Y498 | OH-HH | 43.2 |
| 16 |  |  |  |  |  |  |  | |  |  |  | R13 | NH2-HH21 | D43 | OD2 | 22.0 | D7 | OD1 | Y498 | OH-HH | 25.7 |
| 17 |  |  |  |  |  |  |  | |  |  |  | R13 | NH2-HH21 | H331 | O | 83.4 | D7 | OD1 | K489 | NZ-HZ1 | 36.5 |
| 18 |  |  |  |  |  |  |  | |  |  |  | H14 | ND1-HD1 | A332 | O | 15.0 |  |  |  |  |  |
| 19 |  |  |  |  |  |  |  | |  |  |  | Q15 | OE1 | Q259 | NE2-HE21 | 44.9 |  |  |  |  |  |
| 20 |  |  |  |  |  |  |  | |  |  |  | Q15 | OE1 | K489 | NZ-HZ1 | 23.9 |  |  |  |  |  |
| 21 |  |  |  |  |  |  |  | |  |  |  | Q15 | OE1 | Y498 | OH-HH | 13.0 |  |  |  |  |  |
